# Assessment of Anesthetic Modalities in Otologic Surgery

**DOI:** 10.1097/ONO.0000000000000075

**Published:** 2025-07-14

**Authors:** Phuong H. Bao, David R. Friedland, Jazzmyne A. Adams, Julie K. Freed, Masoud Khani, Jake Luo

**Affiliations:** 1 Department of Otolaryngology and Communication Sciences, Medical College of Wisconsin, Milwaukee, Wisconsin; 2 Caruso Department of Otolaryngology-Head and Neck Surgery, University of Southern California, Los Angeles, CA; 3 Department of Anesthesiology, Medical College of Wisconsin, Milwaukee, Wisconsin; 4 Health Care Informatics, Zilber College of Public Health, University of Wisconsin-Milwaukee, Milwaukee, Wisconsin.

**Keywords:** Anesthesia, Cochlear implant, Surgery, TIVA

## Abstract

**Objective:**

Otologic surgery has specific anesthetic requirements such as avoiding nitrous oxide and allowing facial nerve monitoring, but lacks clear criteria for an optimal anesthetic regimen, often relying on anesthesiologist preference.

**Study Design:**

This study is a retrospective review of 600 primary cochlear implant surgeries and anesthetic variables.

**Setting:**

This study was conducted in a tertiary academic medical center.

**Methods:**

Univariate, multivariate, and cluster analyses of anesthetic regimen association with clinical metrics of postoperative recovery.

**Results:**

Among 600 cochlear implant surgeries, anesthesia regimens included balanced (combination of gas and intravenous agents) (84.3%), gas alone (13.5%), and total intravenous anesthesia (TIVA) (2.2%). By univariate analysis, emergence from anesthesia was shortest with TIVA (11.9 ± 4.6 minutes) and longest with gas (14.2 ± 5.3 minutes), although not reaching statistical significance. Univariate analyses also failed to show a significant correlation between anesthesia regimen and phase I recovery or phase II duration. Multivariate regression indicated significantly shorter emergence times with TIVA compared with gas alone (coeff: −5.29, *P* = 0.0027). Cluster analysis identified 3 groups based on relative remifentanil and gas usage. Patients in cluster 1 (low gas and high remifentanil) had significantly longer emergence times than those in clusters 2 (low gas, low remifentanil: 16.26 ± 5.96 vs 13.39 ± 5.30 minutes; *P* = 0.001) and 3 (high gas, low remifentanil: 16.26 ± 5.96 vs 13.47 ± 5.48 minutes; *P* = 0.0069). Cluster 1 also had longer phase 1 recovery times compared with clusters 2 (65.33 ± 28.87 vs 54.33 ± 25.36 minutes; *P* = 0.0085) and 3 (65.33 ± 28.87 vs 56.38 ± 20.81 minutes; *P* = 0.0365).

**Conclusion:**

TIVA anesthetic regimen is associated with shorter emergence time than gas alone, although the difference in time is small. Balanced regimens are most used among anesthesiologists, and limiting remifentanil dosage may shorten emergence and recovery times.

Otologic surgeries are common interventions for conditions such as hearing loss, infection, trauma, and neoplasm. For example, it has been estimated that over 736,900 cochlear implants (CI) have been implanted worldwide (1). Despite the frequency of otologic surgery, there is little evidence as to which anesthetic regimen provides the best outcomes for patients. This is particularly relevant to otologic surgery given the need to avoid muscle relaxants for facial nerve monitoring, the aged population of many otologic surgery patients (eg, CI), and the avoidance of nitrous oxide due to effects on middle ear pressure, and the risk of dizziness and nausea (2–4).

Inhalational anesthesia (gas), total intravenous anesthesia (TIVA) regimens, or a combination of the 2 (balanced) are the 3 main types of general anesthesia that can be administered during otologic surgery. Inhalational anesthetics are typically used for maintenance of general anesthesia by allowing a loss of awareness and insensitivity to pain. Nitrous oxide (N_2_O), sevoflurane, isoflurane, and desflurane are the most commonly used inhalational anesthetics. TIVA can be used for induction and maintenance as well. Commonly used intravenous anesthetics include, but are not limited to, propofol, etomidate, and ketamine. Many practices also incorporate adjunct medications to complement TIVA including the narcotic remifentanil and dexmedetomidine, an alpha agonist that elicits sedative properties. It is not uncommon for inhalational anesthetics to be used in conjunction with intravenous anesthetics to provide a “balanced” anesthetic which allows for a reduction in the total amount of volatile anesthetic required to achieve an anesthetic state (5,6).

Some studies have compared the efficacy of these anesthetic regimens in nonotologic surgeries. A prospective, single-blind randomized study on postoperative recovery in off-pump coronary artery bypass surgery patients done by Kang et al (7) assessed the patients’ postoperative physical comfort, emotional state, physiological support, physical independence, pain, nausea, and vomiting. The study found no difference in the postoperative recovery and hemodynamic parameters when comparing patients who received TIVA with those who received balanced anesthesia using sevoflurane. Soliz et al (8) found TIVA to be associated with lower grade postoperative complications compared with volatile gas anesthesia in rhinoplasty patients. Talih et al (9) found TIVA to be associated with shorter early emergence times, less bleeding, higher surgeon satisfaction, and lower emergence agitation scores when compared with gas anesthesia, specifically low-flow sevoflurane. Relevant to otology, Jellish et al (10) found that overall recovery was faster with TIVA (e.g., PONV and emergence time) after otologic procedures.

There is a lack of evidence regarding the postoperative recovery of otologic surgery patients stratified by anesthetic regimen. Therefore, we evaluated patients who underwent CI surgery to compare outcomes of gas, TIVA, and balanced anesthetic regimens. We used CI surgery as a surrogate for otologic surgery due to the ability to obtain a large cohort having similar surgical intervention and with relatively consistent durations of anesthetics. For outcomes, we assessed emergence time, time in recovery, postoperative nausea, and immediate postoperative pain medication use to identify best anesthetic practices for otologic procedures.

## METHODS

OTO Clinomics is an IRB-approved comprehensive outcomes measurement platform within our department (IRB#00045896). Using this platform, and the clinical research data warehouse of our NCATS CTSA-supported hub (UL1TR001436), we identified all CI surgeries over a 10-year period by CPT code 69930. Data were filtered for a single surgeon to ensure consistency of technique and to minimize variability in surgical time. Charts were reviewed to ensure the availability of intraoperative anesthesia records and recovery room documentation. Ultimately, this retrospective cohort study analyzed data on 600 patients undergoing CI surgery between June 2013 and June 2023.

Anesthetic agent, dose, emergence time, time in recovery, time in phase II, postoperative nausea, and pain medication were collected for each patient. For anesthesia dose, the highest percent inspired for gas anesthetics and the total dose (mg or mcg) for intravenous (IV) anesthetics were recorded. Emergence time is the time at which the patient regains full consciousness from anesthesia. Time in phase I recovery, the period immediately after surgery within the postanesthesia care unit, was noted. Patients are then transitioned to phase II to prepare them for discharge, and this time was also noted. The number of doses of nausea and pain medications in phase I were included in the data. In addition, information related to patient demographics and other surgical factors were collected. Age at the time of operation, race, and zip code were compiled. Other surgical factors included surgery time, emergence time, whether there was a certified registered nurse anesthetist and/or resident present.

Patients were organized into 3 groups based on their anesthetic regimen: gas, TIVA, and balanced. Univariate analyses were performed by 2-tailed Student *t*-test and chi-square analyses. A multivariate linear regression model, comparing anesthetic regimen with emergence time, time in recovery, and time in phase II, was developed. A StandardScaler was used to preprocess the data to a standardized unit based on mean and standard deviation of the variable. Categorical variables were analyzed relative to a reference variable for that category (eg, TIVA and balanced regimens compared with gas as the reference). Statistically significant associations were noted by a *P* value <0.05 with the coefficient indicating the degree of change in the outcome (eg, emergence time) for every unit change in the studied variable, holding other variables constant. All statistical processing and analyses were performed using R statistical tool (v4.4).

Most patients received a balanced anesthetic regimen, and therefore, the data for this group of patients was further analyzed and disaggregated using a K-means cluster analysis, characterizing patients by relative levels of gas and remifentanil administration. K-means clustering was optimized by silhouette score to select a cluster pattern with least overlap. Ultimately, clustering into 3 groups demonstrated the highest silhouette score (0.557) and was used for further analyses. Orange data mining software (v3.36.2) was used for cluster creation and to obtain feature statistics.

## RESULTS

There were 600 patients with sufficient perioperative data meeting inclusion. Most patients received a balanced anesthetic regimen (84.3%) with 13.5% of patients receiving solely a volatile anesthetic for maintenance of anesthesia, and 2.1% receiving TIVA regimen. The cohort was 52.83% male, which was similar between balanced and gas regimens, although a preponderance of those receiving TIVA were female (Table 1). Other demographic and clinical features were similar between groups. For the entire cohort, the mean surgical time was 72.09 ± 18.53 minutes, and the mean emergence time was 13.68 ± 5.40 minutes.

**TABLE 1.** Demographic characteristics by anesthetic regimen

	Balanced	Gas	TIVA
Mean age (years)	64.5 ± 16.9	66.8 ± 17.5	63.5 ± 16.3
Sex (n = 600)	n =506	n = 81	n = 13
Female	235 (46.4%)	38 (46.9%)	10 (76.9%)
Male	271 (53.6%)	43 (53.1%)	3 (23.1%)
Race			
White	464 (91.6%)	76 (93.8%)	13 (100%)
Black	15 (3.0%)	4 (4.9%)	0 (0%)
American Indian	0 (0%)	1 (1.2%)	0 (0%)
Asian	9 (1.8%)	0 (0%)	0 (0%)
Multiracial	3 (0.6%)	0 (0%)	0 (0%)
Other	12 (2.4%)	0 (0%)	0 (0%)
Patient refused	3 (0.6%)	0 (0%)	0 (0%)
Ethnicity			
Hispanic	11 (2.2%)	1 (1.2%)	0 (0%)
Non-Hispanic	491 (97.0%)	80 (98.8%)	13 (100%)
Patient refused	3 (0.6%)	0 (0%)	0 (0%)
Unknown	1 (0.2%)	0 (0%)	0 (0%)
ASA Class			
I	24 (4.7%)	4 (4.9%)	0 (0%)
II	209 (41.3%)	32 (39.5%)	5 (38.5%)
III	256 (50.6%)	43 (53.1%)	8 (61.5%)
IV	17 (3.4%)	2 (2.5%)	0 (0%)
Mallampati			
I	131 (25.9%)	29 (35.8%)	3 (23.1%)
II	256 (50.6%)	41 (50.6%)	6 (46.2%)
III	89 (17.6%)	5 (6.2%)	2 (15.4%)
IV	8 (1.6%)	3 (3.7%)	0 (0%)
Mean surgical time (min)	72.69 ± 19.08	68.75 ± 15.29	69.54 ± 12.76
Mean emergence time (min)	13.65 ± 5.44	14.15 ± 5.26	11.92 ± 4.65
Mean time in recovery (Min)	55.69 ± 24.96	59.49 ± 21.3	57.77 ± 26.14
Mean time in phase II (min)	124.64 ± 56	138.1 ± 70	134.42 ± 86
Anesthesia staffing			
Anesthesiologist alone	40 (7.9%)	7 (8.6%)	3 (23.1%)
With resident	187 (37.0%)	42 (51.9%)	4 (30.7%)
With CRNA	276 (54.5%)	32 (39.5%)	6 (46.2%)
With CRNA and resident	3 (0.6%)	0 (0%)	0 (0%)

ASA Class indicates American Society of Anesthesiologists Physical Status Classification System; CRNA, certified registered nurse anesthetist.

**FIG. 1.**
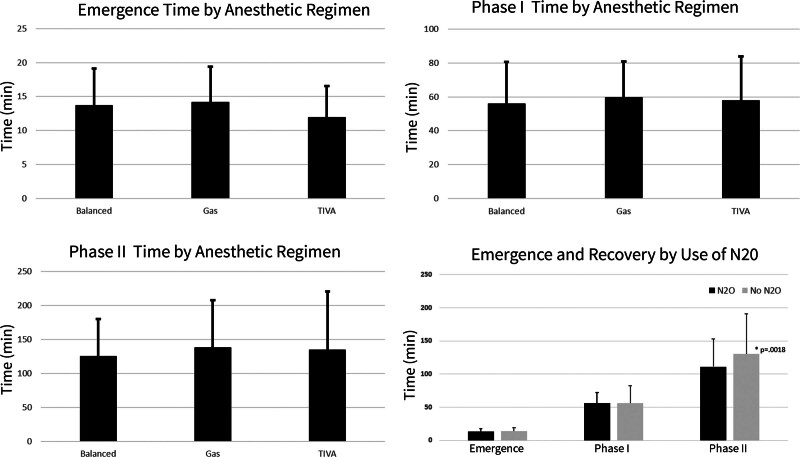
Emergence, phase I recovery, and phase II recovery times by anesthetic regimen and by the use of nitrous oxide. Those receiving nitrous oxide had significantly shorter phase II recovery times.

By univariate analysis, there was no significant difference in the average time of emergence, time spent in phase I, and time spent in phase II relative to anesthetic regimen (Fig. 1). Although the use of N_2_O is typically contraindicated in otologic surgery, 112 of 600 patients had administration of N_2_O during their surgery. This group had significantly shorter duration of time in phase II of recovery than patients who did not receive nitrous oxide (111 ± 42 vs 130 ± 61 minutes, *P* = 0.0018).

**FIG. 2.**
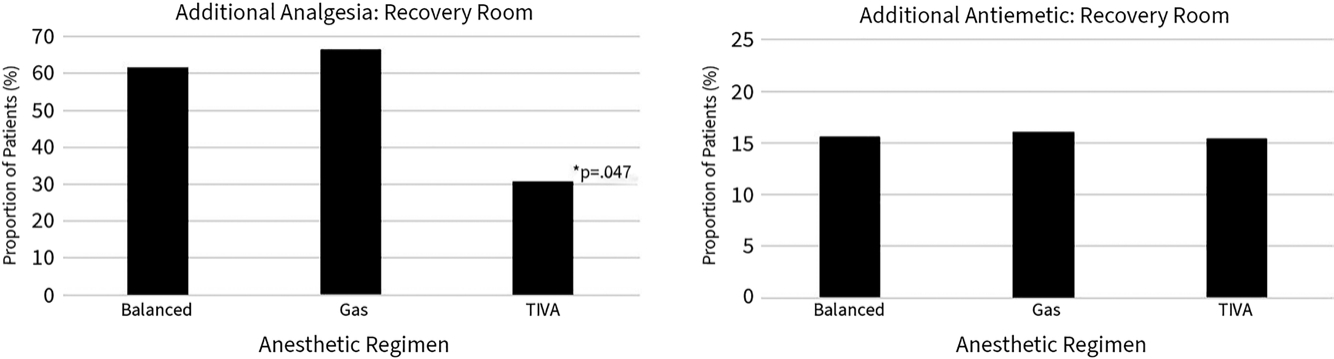
Use of analgesics and antiemetics in the recovery room by anesthetic regimen. Use of TIVA significantly reduced analgesic need in the recovery room.

Only 30.8% of patients having TIVA anesthetic required additional analgesia in the recovery room compared with over 60% of patients having a gas or balanced regimen (Fig. 2). These results were significant by chi-square analysis (*X*^*2*^(2, N = 600)=6.12, *P* = 0.047). Regardless of anesthetic regimen, only about 1 in 7 patients required antiemetics in the recovery room. There was no statistically significant difference in antiemetic usage by regimen.

Multiple linear regression (Table 2) demonstrated a statistically significant association with longer emergence time with any incorporation of sevoflurane (coefficient=0.48, *P* = 0.0277), remifentanil (mg) (coefficient = 0.89, *P* = 0.0002), and propofol (mg) (coefficient=1.34, *P* = 0.0001) in the anesthetic regimen. Furthermore, TIVA regimen was found to be associated with a shorter emergence time than gas alone (coefficient = −5.29, *P* = 0.00273). Multivariate analyses also demonstrated that emergence time in cases that included a resident in-training (15 ± 5.93 minutes, reference) was significantly longer than cases in which an anesthesiologist worked alone (12.5 ± 4.29, *P* = 0.00607) or with a certified registered nurse anesthetist (13 ± 4.78, *P* = 0.00145).

**TABLE 2.** *Multivariate linear regression model for predictors of emergence time (min*)

Feature	Coefficient	*P* value
Age	**0.689934**	**0.00547**
Sex		
Male	Reference	Reference
Female	0.345179	0.44334
Race		
White	Reference	Reference
Black	0.963253	0.42747
Asian	−0.319017	0.85671
AI/AN	6.795315	0.19802
Multiracial	−1.482165	0.57524
Other	−0.032552	0.98307
Patient refused	3.453996	0.25671
Surgical time (min)	0.191375	0.40776
Anesthetics		
** **Sevoflurane	**0.48117**	**0.0277**
Desflurane	0.21177	0.3491
Isoflurane	−0.228373	0.2856
N_2_O	−0.302097	0.15783
Fentanyl (mcg)	−0.048108	0.83312
Remifentanil (mg)	**0.889921**	**0.0002**
Propofol (mg)	**1.342858**	**0.0001**
ASA Class		
I	Reference	Reference
II	−0.196766	0.85582
III	−0.181342	0.8705
IV	1.971786	0.23268
Mallampati score		
I	Reference	Reference
II	0.40429	0.41648
III	0.985417	0.14539
IV	2.834763	0.08736
Anesthesia staffing		
Resident+staff	Reference	Reference
Resident+CRNA+staff	0.644899	0.83192
CRNA+staff	**−1.498205**	**0.00145**
Staff only	**−2.250456**	**0.00607**
Anesthetic Regimen		
Gas	Reference	Reference
** **TIVA	**−5.289304**	**0.00273**
Balance	−0.000439	0.99949

Bold-faced values indicate statistically significant. Positive coefficients indicate association with longer emergence times; negative coefficients with shorter emergence time.

ASA Class indicates American Society of Anesthesiologists Physical Status Classification System; CRNA, certified registered nurse anesthetist; TIVA, total intravenous anesthesia.

**FIG. 3.**
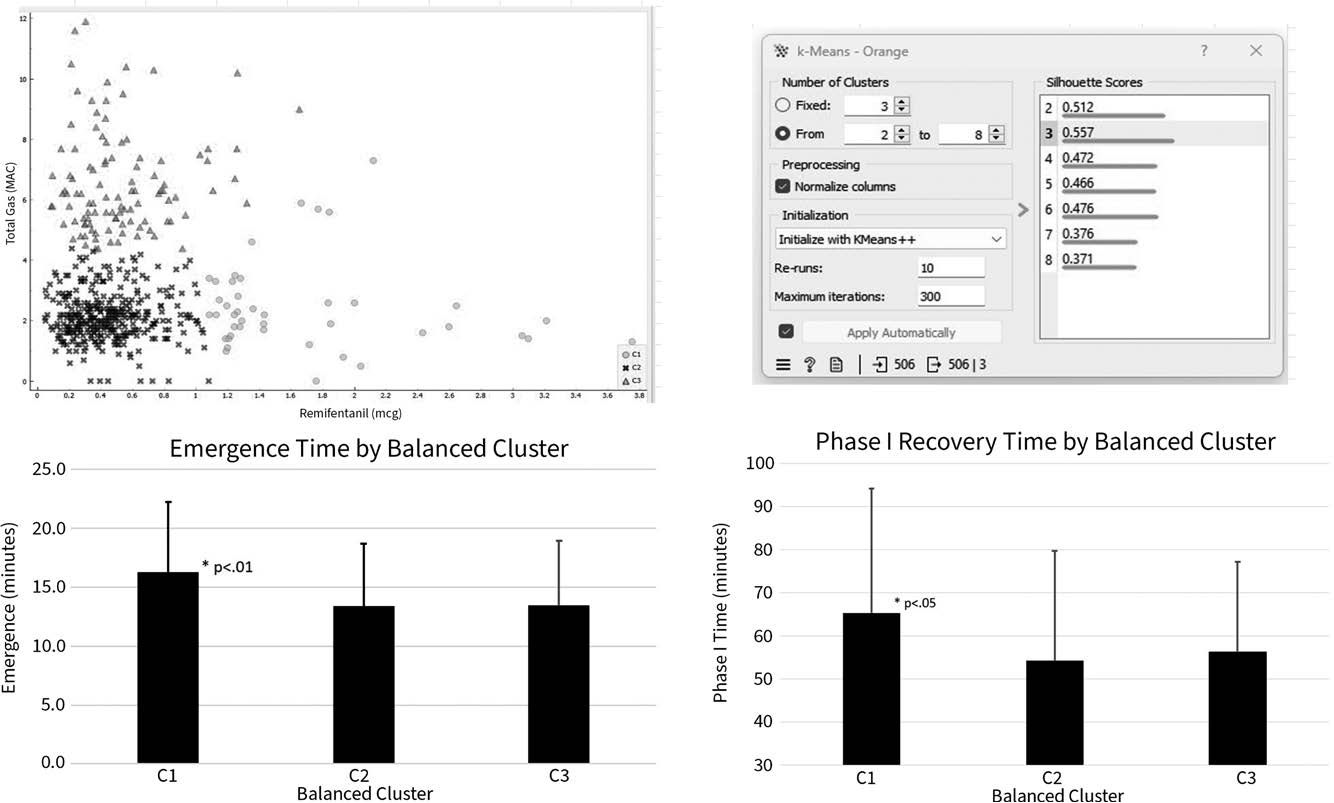
Balanced anesthetic cluster analysis. Cluster C1, relatively high dosage of remifentanil, in combination with volatile anesthetics significantly prolonged emergence and phase I recovery time.

Cluster analysis of the balanced group identified 3 clusters relative to amounts of remifentanil and gas usage: (1) low gas/high remifentanil (c1, n = 43), (2) low gas/low remifentanil (c2, n = 358), and (3) high gas/low remifentanil (c3, n = 105) (Fig. 3). Those in cluster 1 (low gas, high remifentanil) had significantly longer emergence times than those in clusters with low remifentanil: cluster 2 (16.26 ± 5.96 vs 13.39 ± 5.30 minutes; *P* = 0.001) and cluster 3 (16.26 ± 5.96 vs 13.47 ± 5.48 minutes; *P* = 0.0069). Similarly, those in cluster 1 (low gas, high remifentanil) had significantly longer phase 1 recovery times than those in cluster 2 (65.33 ± 28.87 vs 54.33 ± 25.36 minutes; *P* = 0.0085) or those in cluster 3 (65.33 ± 28.87 vs 56.38 ± 20.81 minutes; *P* = 0.0365).

Further analyses were done to assess for fentanyl related hyperalgesia, which can prolong recovery times and require additional doses of pain medication. Only 12 of the 600 patients remained in the recovery room more than 2 standard deviations from the mean. Among these, 9 had a balanced anesthetic, 2 had volatile gases only, and 1 had TIVA (similar proportions to the overall cohort). Remifentanil was used in 9 of these 12 patients, and there was no statistically significant difference in recovery room time between those receiving remifentanil and those not (83 vs 56 minutes; *P* = 0.0893), although the sample size was small. We also assessed for the use of ketamine, which has been reported to mitigate such hyperalgesia. Ketamine was used in 23 of the 600 patients. Among those with and without ketamine, time in phase I recovery (52.8 vs 56.4 minutes; *P* = 0.501) and time in phase 2 recovery (121.6 vs 126.7 minutes; *P* = 0.730) were similar. While propofol can also mitigate fentanyl induced hyperalgesia, 97.7% of our cohort received propofol and therefore no further analysis was performed.

## DISCUSSION

We found that TIVA was associated with faster emergence time than gas or balanced anesthesia regimens with multivariate analysis. TIVA was also associated with less immediate postoperative analgesia usage. However, balanced regimens appeared to be used most often, and in these cases, lower doses of remifentanil were associated with faster emergence. In addition, although N_2_O is typically contraindicated in otologic surgeries, it was associated with shorter time spent in phase II despite no difference in emergence time or time spent in phase I.

As previously mentioned, several studies have looked at differences in postoperative recovery for patients receiving TIVA versus gas anesthesia (8–10). These studies have found TIVA to be associated with better recovery through several parameters including reduced risk of postoperative nausea and vomiting, shorter emergence times, less bleeding, high surgeon satisfaction, and lower emergence agitation scores. Nadstawek et al compared the recovery rate between 2 groups: (1) TIVA with propofol and alfentanil and (2) gas with N_2_O and enflurane. The TIVA group demonstrated faster recovery (30–40 minutes) for simple and discriminating motor activities and short and long-term memory compared with the gas group (80 minutes). Speech-related functions were inhibited in the gas group (11). While we did not evaluate functional recovery, for otologic surgery, the TIVA regimen was similarly found to be associated with a shorter emergence time. However, when similar IV agents are used in relatively high doses along with gas, we saw longer emergence time and time in phase 1. This finding seems to contradict our own findings and reports on TIVA and its association with better recovery, suggesting an interaction between IV and gas agents that needs to be better accounted for as relates to emergence from anesthesia.

Nitrous oxide is contraindicated in most otologic surgeries, particularly those traversing the middle ear as nitrous oxide is more soluble than nitrogen, thus replacing it in the middle ear causing increased pressure and volume (12). Past studies have demonstrated use of nitrous oxide can generate enough pressure to result in the opening of the Eustachian tube (13,14). Despite this contraindication, nitrous oxide was used in almost 20% of cases (n = 112) included in our study. There was no significant difference in time of emergence and time in phase I with or without nitrous oxide use although phase II was shorter. While nitrous oxide has advantages such as faster induction, lower respiratory and hemodynamic effects, and cost effectiveness, the lack of significant improvement in outcomes in our otologic patients suggests that the risk to the surgical field should outweigh the perceived anesthetic benefits and should not be used.

Indeed, other evidence shows anesthetic disadvantages to the use of nitrous oxide, perhaps facilitating communication with anesthesiologists to avoid its use in otologic surgery. For example, nitrous oxide usage is associated with postoperative nausea and vomiting. A meta-analysis completed by Divatia et al (15) concluded avoiding nitrous oxide reduced the odds of postoperative nausea and vomiting by 37%. In a randomized control trial, Myles et al (16) reported that avoiding nitrous oxide decreased the incidence of postoperative complications after major surgeries. It is worthy to note that sometimes N_2_O appears on the anesthesia machine despite it not being used which, in our institution, then gets incorporated into the electronic health record. This is an issue with the infrared monitoring system within the machine.

In the postoperative period, for an outpatient surgery such as CI surgery, uncontrolled pain or nausea may prolong a patient’s stay. Visser et al (17) reported TIVA was associated with less postoperative nausea compared with gas anesthesia. Our study did not show a statistically significant difference in nausea by regimen. This may be due to low sample size and occurrence as only 1 in 7 CI patients required antiemetics. In addition, most patients in the Visser study had concurrent muscle relaxants, and many surgeries were of longer duration than ours, factors that may impact postoperative nausea.

Current reports are contradictory regarding the effects of TIVA on postoperative pain. Studies such as those completed by Wong et al (18) and Soliz et al (19) have found TIVA to be associated with less postoperative pain. Contrastingly, Lenartova et al (20) found TIVA to not be associated with a decrease in postoperative pain or reduced pain medication usage. While these past studies examined a wide range of surgeries, none looked at TIVA and its influence on postoperative pain for otologic surgery. The findings of our study provide evidence supporting TIVA and its association with improved postoperative pain management for otologic cases.

Of note, the use of fentanyl products has been reported to paradoxically cause rebound hyperalgesia, which can require additional pain medication during recovery and delay discharge. This may be related to rapid cutoff of fentanyl infusion, something we did not specifically measure in this study (21). Propofol is often used in combination with remifentanil because preloading with propofol is reported to mitigate remifentanil-induced hyperalgesia (22). Almost 98% of our patients received loading with propofol, and this may account for the fact that only 12 of 600 patients had severely prolonged phase I recovery. Regardless, judicious use of fentanyl should be considered to prevent such rebound effect.

Even though our clinical differences were minor, it may be of interest to reduce gas anesthetic usage during otologic procedures to reduce greenhouse gas emissions. Most volatile anesthetics are ozone depleters, greenhouse gases, or both. About 95% of these volatile anesthetics are exhaled unchanged during anesthesia, which are then ultimately released into the atmosphere. This has long-term negative implications on climate change when considering the lifetime of these volatile gases. Chlorofluorocarbons, such as isoflurane and halothane, have a lifetime of 50–100 years; desflurane has a lifetime of 8.9–21.0 years (23). Sherman et al (24) previously suggested that desflurane, nitrous oxide, and other gas anesthetics should only be used if these gas anesthetics will reduce morbidity and mortality compared with alternative drugs. Based on our results, patients who received the gas regimen did not show a faster recovery rate and had no clear recovery advantages. As such, gas anesthetics should have limited usage in the context of climate change, given no auxiliary advantage to patient recovery.

With all the existing clinical literature in support of TIVA and its benefits of better postoperative recovery, it is important to consider why anesthesiologists still use volatile anesthetics. Wong et al (25) sought to answer this question and found that among infrequent users of TIVA, the main barriers to TIVA use were issues such as additional effort, institutional preference, lack of real-time monitoring of propofol concentration, risk of missing drug delivery failure, and increased turnaround time. Usage of volatile anesthetics is more precise and simpler as the anesthesia delivery system can calculate, based on age, the minimum alveolar concentration (MAC) that is achieved with the end tidal of the gas. Whereas there is no end-tidal concentration to measure when using TIVA, making dosing and monitoring more difficult. These are all factors to take into consideration for future studies to make findings even more robust and conclusive.

There are several limitations to this study. The study is a retrospective chart review and, therefore, does not have matched controls for which anesthetic regimen patients received. We had a notably uneven number of patients who received each anesthetic regimen, particularly a small number who received TIVA. Future studies should include prospective randomized enrollment to equate the numbers of patients receiving each anesthetic regimen or interrogate a larger cohort to allow for matching anesthetic regimen across demographics. We relied on data recorded on the patient’s electronic and anesthesia record, which could be poorly documented or contain missing data. In addition, we did not record the timing of administration of intravenous medications such as propofol and remifentanil, which could impact emergence time, time in recovery, and postoperative use of antiemetics and supplemental pain medication. Furthermore, while the patients were standardized by the type of otologic surgery, the surgeon, and the surgical setting, patients had varying BMIs and comorbidities that may influence anesthetic metabolism and postoperative outcomes. Finally, while we used CI surgery as a surrogate for otologic procedures, inclusion of those with middle ear dissection or longer anesthetic times would be necessary to demonstrate generalizability.

## CONCLUSION

TIVA anesthetic regimen showed shortened emergence time than those using volatile gases, although the difference was small. Patients who received a balanced anesthetic regimen with relatively higher doses of remifentanil had significantly longer emergence and phase 1 recovery times. No distinct advantages in recovery were noted for any regimen. Given the environmental impact of volatile gas anesthetic agents, IV-based anesthesia for otologic surgeries should be considered.

## FUNDING SOURCES

This OTO Clinomics project was funded through the Advancing a Healthier Wisconsin Endowment at the Medical College of Wisconsin with support by the National Center for Advancing Translational Sciences, National Institutes of Health, Award Number UL1TR001436. The content is solely the responsibility of the authors and does not necessarily represent the official views of the NIH.

## CONFLICT OF INTEREST STATEMENT

None declared.
